# Whipple's Disease Presenting With a Chief Complaint of Dyspnea and Cough From Pulmonary Invasion Without Evidence of Gastrointestinal Involvement

**DOI:** 10.7759/cureus.54554

**Published:** 2024-02-20

**Authors:** Michael Ladna, John George, Christopher E Forsmark

**Affiliations:** 1 Internal Medicine, University of Florida College of Medicine, Gainesville, USA; 2 Gastroenterology, University of Florida College of Medicine, Gainesville, USA

**Keywords:** interstitial lung disease, chronic lung disease, immunosuppression, tropheryma whipplei, whipple’s disease

## Abstract

A patient with immune thrombocytopenia, systemic lupus erythematosus on chronic corticosteroids, and interstitial lung disease was referred to the pulmonology clinic due to progressively worsening dyspnea. A bronchoscopy was done and a thorough workup was negative for any infectious pathology or malignancy. A lung biopsy with MicroGenDX test (MicroGen Diagnostics, Lubbock, TX) revealed *Tropheryma whipplei*, consistent with a Whipple disease diagnosis. Histopathology of biopsy specimens from an esophagogastroduodenoscopy showed moderate chronic active *Helicobacter* gastritis and unremarkable duodenal specimens without evidence of *Tropheryma whipplei*. For *Helicobacter pylori* gastritis, she was prescribed quadruple therapy with omeprazole, bismuth, metronidazole, and tetracycline. For pulmonary Whipple's disease, she completed two weeks of IV ceftriaxone, which led to improvement in dyspnea, and then was transitioned to 12 months of oral sulfamethoxazole-trimethoprim. In rare cases, Whipple's disease can present as isolated pulmonary disease without gastrointestinal involvement, especially in immunosuppressed patients with compromised lungs.

## Introduction

Whipple’s disease tends to present with gastrointestinal (GI) manifestations, such as abdominal pain, diarrhea, and weight loss, due to the involvement of the small bowel, typically the duodenum [1-3]. It is important for clinicians to recognize that Whipple's is a multi-systemic disease that can involve the central nervous system (CNS) and lungs [4]. Due to the involvement of numerous organs, Whipple’s disease can present with under-recognized symptoms, such as cough and dyspnea. It is also important to recognize that pulmonary manifestations can precede the involvement of the GI tract [5] and the lack of *Tropheryma whipplei* in GI tract histopathology does not rule out a diagnosis of Whipple’s disease.

## Case presentation

**Figure 1:**
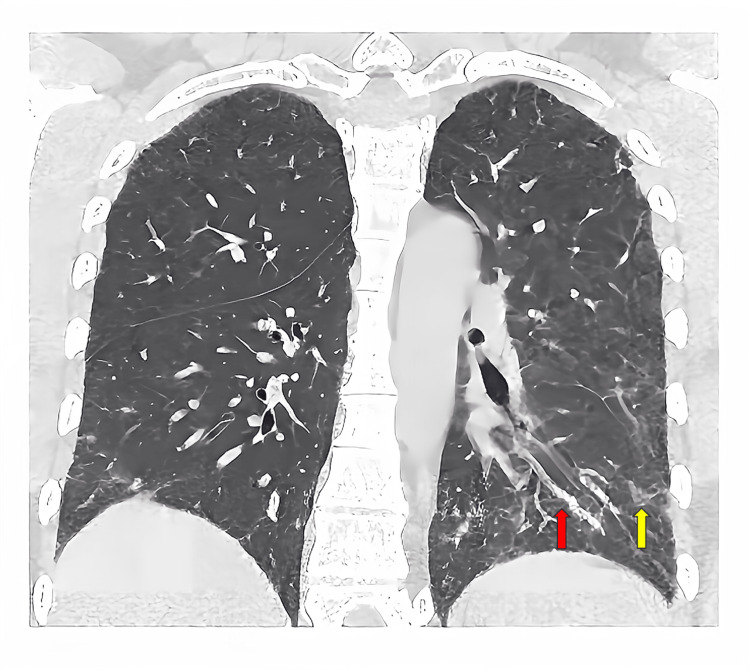
High-resolution CT of the chest showing subpleural and peribronchovascular bilateral reticulations (red arrow) predominately in lower lobes and lingula with mild ground glass opacities and traction bronchiectasis (yellow arrow) without honeycombing, cavitation, or effusions as well as enlargement of the main pulmonary artery concerning for pulmonary hypertension.

An African American female in her early 40s with a past medical history of immune thrombocytopenia (ITP), systemic lupus erythematosus (SLE) on chronic corticosteroids for several years, and interstitial lung disease (ILD) most consistent with nonspecific interstitial pneumonitis (NSIP) presented to her primary care physician (PCP) with several weeks progressively worsening dyspnea, cough, and arthralgias. She was referred to a pulmonologist and underwent a bronchoscopy with bronchoalveolar lavage (BAL) and lung biopsy as an outpatient. BAL bacterial, acid-fast bacilli, and fungal cultures did not yield any growth, Aspergillus galactomannan of the BAL was negative, BAL cytology was negative for malignancy, BAL *Pneumocystis jirovecii* polymerase chain reaction (PCR) was negative, hypersensitivity pneumonitis panel was negative, and BAL pneumonia PCR panel was negative for any infectious organism. The lung biopsy revealed peribronchiolar chronic inflammation with multinucleated giant cells and organizing pneumonia-like changes. Although a periodic acid-Schiff (PAS) stain was negative, MicroGenDX (MicroGen Diagnostics, Lubbock, TX), which is a DNA PCR test, revealed 26% *Tropheryma whipplei*. Given her progressively worsening symptoms and MicroGenDX results, a diagnosis of Whipple's disease was made. High-resolution computed tomography (CT) scan of the chest showed subpleural and peribronchovascular bilateral reticulation predominately in lower lobes and lingula, with mild ground glass opacities and traction bronchiectasis without honeycombing, cavitation, or effusions (Figure 1).

**Figure 2:**
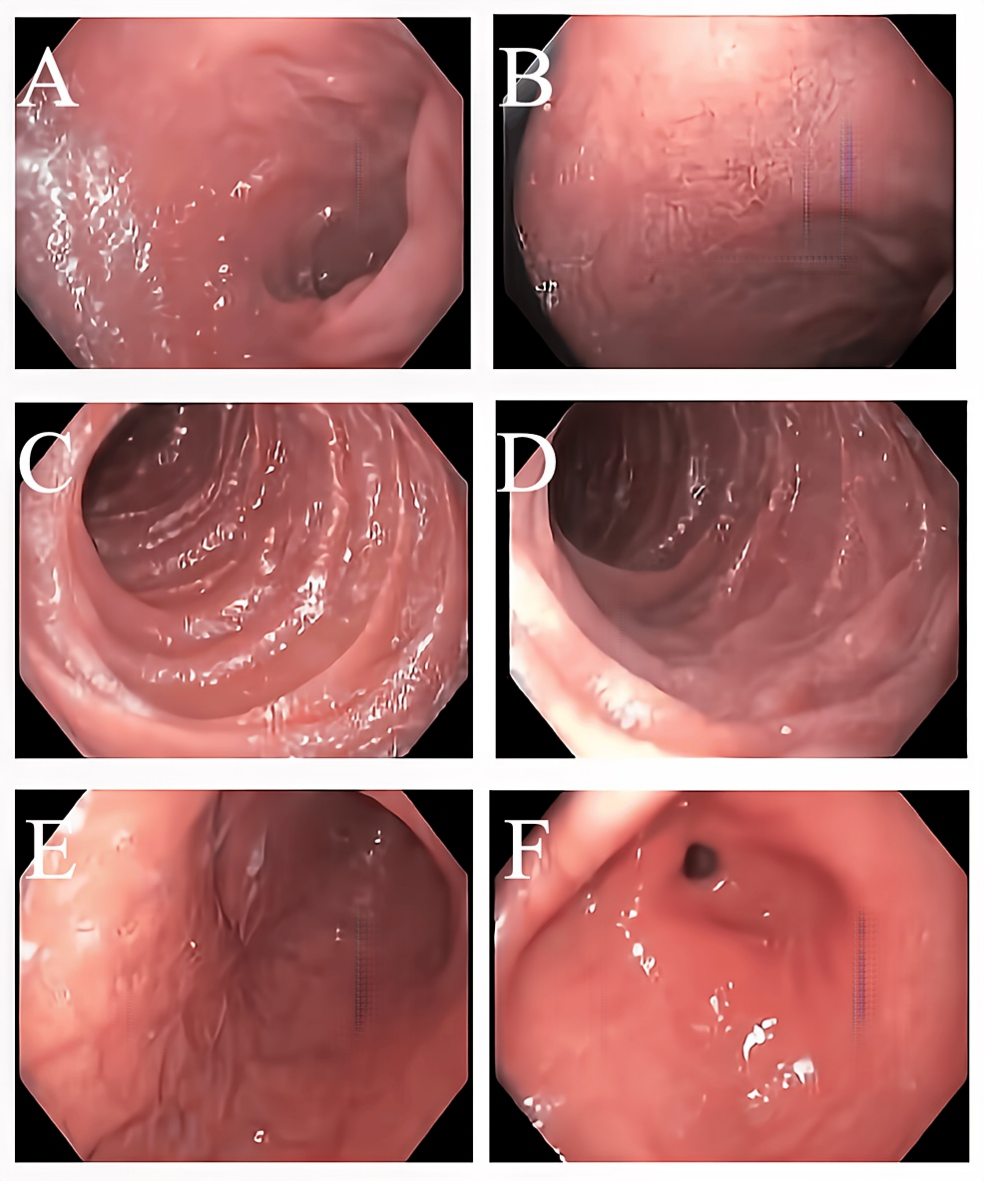
Esophagogastroduodenoscopy showing mild diffuse erythematous mucosa in the stomach at antrum (E) and pre-pylorus (F) without evidence of active or recent bleeding and normal appearing duodenal mucosa (A-D).

There was also an enlargement of the main pulmonary artery concerning for pulmonary hypertension. This morphologic pattern was most consistent with NSIP. Due to the diagnosis of Whipple’s disease, she was admitted to the hospital for initiation of IV antibiotics and workup for intestinal manifestations via an esophagogastroduodenoscopy (EGD), which revealed mild diffuse erythematous mucosa in the stomach without evidence of active or recent bleed and normal appearing duodenal mucosa (Figure 2).

Histopathology of biopsy specimens from the stomach revealed moderate chronic active *Helicobacter* gastritis in antral and oxyntic mucosa without metaplasia or dysplasia while duodenal specimens were unremarkable. PAS stain was negative in EGD biopsy samples.

Infectious disease (ID) was consulted and recommended initiation of ceftriaxone. She reported an allergy to ceftriaxone and thus was upgraded to the medical intensive care unit (MICU) for a challenge test, which consisted of initial administration of 10% of a full 1 g dose, followed by a full 1 g dose. She tolerated both doses of ceftriaxone without evidence of an allergic reaction. A peripherally inserted central catheter (PICC) was placed. She was hospitalized for a total of three days and discharged home with a plan for two weeks of IV ceftriaxone 2 g daily, followed by 12 months of oral sulfamethoxazole-trimethoprim one double strength (DS) tablet twice daily (BID). She presented to the ID clinic one week after discharge and was prescribed a 10-day course of quadruple therapy with omeprazole, bismuth, metronidazole, and tetracycline for the treatment of *H. pylori *gastritis. After completing a two-week course of ceftriaxone, her symptoms improved and she was transitioned to oral sulfamethoxazole-trimethoprim.

## Discussion

Whipple's disease is a rare, chronic, systemic, granulomatous infectious pathology caused by the bacterium* Tropheryma whipplei *[6]. It has a prevalence of approximately one per 1,000,000 patients and an annual incidence between one and six per 10,000,000 patients [7]. The mean age of diagnosis is 55 years of age with a male predominance, with 85% of cases being found in men [7]. The disease is most common in white ethnicity, very rare in the Asian population, and virtually unknown in the African population [4]. Recent data show the prevalence in female patients has been increasing [2].

Actinobacteria such as *Tropheryma whipplei* are found in soil, freshwater, or seawater sediments, with 37-66% of sewage plant influxes having evidence of *Tropheryma whipplei* [8,9]. Exposure to contaminated soil has been hypothesized to be a potential route to infection with farmers being at especially high risk [4]. Although a rare disease, asymptomatic carriage of *Tropheryma whipplei* is relatively common with 4% of normal controls and 12% of asymptomatic high-risk patients such as sewer workers testing positive for *Tropheryma whipplei* [10].

The most common presentation of Whipple’s disease exhibits predominantly GI symptoms with infection occurring in the small intestine, particularly the duodenum, and is known as classic Whipple's disease (CWD). The most common symptoms of Whipple's disease are diarrhea, abdominal pain, arthralgia, arthritis, weight loss, low-grade fever, anemia, and adenopathy [1-3]. Severe complications, such as CNS involvement, can also occur but are less common, with a prevalence of around 15% [4]. The typical course of CWD is a prodromal stage of months or even years of arthralgia before the onset of typical GI symptoms [4]. This makes the diagnosis of Whipple’s disease especially difficult. Once diarrhea or weight loss manifests, the mean time to diagnosis is 12 months and eight months from the onset of those symptoms, respectively [11]. The time interval from the prodromal stage to CWD has been shortened due to exposure to immunosuppressive agents such as corticosteroids or antitumor necrosis factor inhibitors [12,13].

The gold standard for diagnosis of Whipple’s disease is via histopathology of duodenal biopsies. Two positive biopsy specimens are required for a diagnosis while two negative biopsy specimens are required to exclude Whipple’s disease. Once a diagnosis is made, PCR of cerebral spinal fluid (CSF) samples is recommended to exclude CNS involvement [4]. It should be noted that even with massive infiltration of the duodenal mucosa, most cases show an unremarkable and nonspecific appearance of the duodenum with pale yellow mucosa and clumsy dilated villi and ectasia of lymph vessels [14,15]. Foamy macrophages containing large amounts of diastase-resistant PAS-positive particles in the lamina propria of the GI tract with Ziehl-Neelsen staining are used to distinguish between non-acid fast *T. whipplei* and acid-fast mycobacteria [4].

Response to treatment tends to be rapid with diarrhea and fevers resolving within days though arthropathy tends to take several weeks to fully recede [3]. Treatment of Whipple’s disease involves an induction period with 14 days of either IV ceftriaxone or IV meropenem, followed by 12 months of maintenance therapy with co-trimoxazole or doxycycline in combination with hydroxychloroquine [4]. Co-trimoxazole was found to be more effective than tetracyclines and had better CNS penetration, making it the preferred first-line agent for maintenance therapy [1]. If left untreated, Whipple’s disease has a fatal course; however, the mortality rate of treated Whipple’s is largely unknown [4]. Even after the successful eradication of *T. whipplei*, PAS-positive macrophages can persist for years and do not necessarily indicate treatment failure [16].

Our case was atypical as it presented with a chief initial complaint of dyspnea with predominant pulmonary involvement that had preceded GI involvement and symptoms. There have been only a handful of cases in the literature documenting Whipple’s disease with pulmonary symptoms preceding any evidence of GI involvement [5]. In addition, her female sex and African American ethnicity were both uncommon in Whipple’s disease, as this is typically a pathology of white males. It is also equally likely that the *T. whipplei* found on lung biopsy did not represent active disease. A case can be made for asymptomatic carriage given the lack of PAS-positive macrophages from the biopsy of the lung in the setting of two underlying pathologies, which could explain her symptomology. ILD, a progressive chronic pulmonary disease, can explain the dyspnea and cough while SLE can explain the arthralgias, especially given her case of treatment-refractory disease requiring chronic corticosteroid usage. Whether the disease was active or not was exceedingly difficult to differentiate in this case, and thus antibiotic therapy was initiated since untreated Whipple’s disease would progress and further damage her already compromised lungs. The improvement in her symptoms with the completion of ceftriaxone gives some credence to this being active Whipple's; however, the placebo effect cannot be discounted.

## Conclusions

Immunosuppression from chronic corticosteroid exposure and pre-existing damage to the lung tissue from underlying ILD were factors that likely contributed to Whipple's disease initially invading pulmonary tissue with pulmonary manifestations preceding gastrointestinal. The clinician should be aware that Whipple’s disease can present with isolated pulmonary manifestations, especially in those with predisposing risk factors such as immunosuppression and chronic lung disease. Treatment consists of two weeks of IV ceftriaxone, followed by 12 months of oral trimethoprim-sulfamethoxazole. Monitoring for effective treatment and recurrence can be difficult since PAS-positive macrophages can persist for years.
